# Circulating MicroRNAs as Biomarkers of Accelerated Sarcopenia in Chronic Heart Failure

**DOI:** 10.5334/gh.943

**Published:** 2021-08-30

**Authors:** Rizwan Qaisar, Asima Karim, Tahir Muhammad, Islam Shah, Javaidullah Khan

**Affiliations:** 1 Basic Medical Sciences, College of Medicine, University of Sharjah, Sharjah, AE; 2 University of Health Sciences, Lahore, PK; 3 Departmenr of Biochemistry, Gomal Medical College, Dera Ismail Khan, PK; 4 Department of Cardiology, Al Qassimi Hospital, Sharjah, AE; 5 Department of Cardiology, Post Graduate Medical Institute, Hayatabad Medical Complex, Peshawar, PK

**Keywords:** Sarcopenia, heart failure, microRNAs, hand-grip strength

## Abstract

**Background:**

Sarcopenia is a critical finding in patients with chronic heart failure (CHF). However, the search for a definitive biomarker to predict muscle and functional decline in CHF remains elusive.

**Objectives:**

We aimed to correlate the circulating levels of selected miRs with the indexes of sarcopenia during healthy aging and in patients with CHF.

**Methods:**

We analyzed the association of circulating microRNAs (miRs) levels including miR-21, miR-434-3p, miR424-5p, miR-133a, miR-455-3p and miR-181a with sarcopenia indexes in male, 61–73 years old healthy controls and patients with CHF (N = 89–92/group).

**Results:**

Patients with CHF had lower hand-grip strength (HGS), appendicular skeletal mass index (ASMI) and physical capacity than healthy controls. Circulating miR-21 levels were higher and miR-181a, miR-133a, miR-434-3p and miR-455-3p levels were lower in patients with CHF than healthy controls. Among the sarcopenia indexes, HGS showed the strongest correlation with miR-133a while ASMI showed the strongest correlations with miR-133a, miR-434-3p and miR-455-3p. Among the miRs, miR-434-3p showed the highest area under the curve in testing for sensitivity and specificity for CHF. These changes were associated with higher expressions of the markers of inflammation, oxidative stress and muscle damage in CHF patients.

**Conclusion:**

Taken together, our data show that circulating miRs can be useful markers of muscle health and physical capacity in the sarcopenic elderly with CHF.

## Introduction

Sarcopenia, the age-related muscle decline is a significant health problem in the elderly. The loss of muscle mass and strength start in the fourth decade of life and progresses at 3–8% per decade [1]. The prevalence of sarcopenia is 5–13% between 60 and 70 years of age and further increases with advancing age, reaching up to 50% in octogenarians [2]. It is also frequently associated with adverse events such as fall, osteoporosis, dependent lifestyle, and mortality.

While sarcopenia is a physiological process, several geriatric comorbidities can accelerate its progression. Among them, chronic heart failure (CHF) is a well-recognized co-morbidity in the elderly. CHF is caused by cardiac contractile or diastolic dysfunction with reduced cardiac output and progressive deterioration of the cardiac contractility. Sarcopenia is frequently associated with CHF, which may further deteriorate cardiac and skeletal muscle dysfunction [3]. The prevalence of sarcopenia is ≈20% higher in the elderly with CHF than healthy individuals [4]. Further, sarcopenia in CHF patients has an early onset, irrespective of cardiac output and requires timely diagnosis and monitoring for proper interventions [3]. However, the search for an effective biomarker to characterize sarcopenia in CHF patients remains elusive.

Micro RNAs (miRs) are small non-coding RNAs, 21–23 nucleotides in length and regulate gene transcription by repressing translation and/or degrading mRNAs [5]. Several reports recognize an essential role of miRs in skeletal muscle health and disease, as aberrant expression of miRs is associated with multiple skeletal muscle diseases including sarcopenia [5,6]. miRs derived from skeletal muscle and other tissues are easily detectable in circulation, indicating their diagnostic potential in sarcopenia. Several studies indicate an altered expression of both the muscle-specific and non-muscle specific miRs in the circulation in the elderly [5,6]. Additionally, the patients with CHF also show significant alterations in the plasma miRs profile [7]. Among the several circulating miRs levels perturbed in sarcopenia and/or CHF, miR-21, miR-181a, miR-133a, miR-424-5p, mir-434-3P and miR-455-3P have emerged as potential candidates for diagnosing and/or monitoring muscle health [8,9]. However, their association with sarcopenia indexes in healthy aging and in patients with CHF as well as their diagnostic potential in CHF are not well characterized. Further, several factors including different detection methods (RT-PCR, microarray, high throughput sequencing), variability in patients’ clinical conditions including age and sex, and limited sample size may account for the discrepancy in the literature which may reduce the diagnostic potential of the miRs. Additionally, systemic oxidative stress and chronic inflammation can also affect generalized health and circulating miRs levels but their correlation with selected miRs in sarcopenia is not known. In this study, we asked whether the circulating levels of selected miRs correlate with the indexes of sarcopenia during healthy aging and in patients with CHF.

## Methods

### Study design & participants

We recruited 181 ambulatory male participants as healthy controls (N = 92) and patients with CHF (N = 89) after obtaining ethical approvals at the University of Health Sciences, Lahore (approval #UHS/ERB/22587/2016), teaching hospital of Gomal Medical College, Dera Ismail Khan (approval #GMC/157/2016) and cardiac rehabilitation center, Hayatabad Medical Complex, Peshawar, (approval #HMC/253/2017), Pakistan. The regional research ethical committees at these university hospitals approved this study. All participants provided written informed consent. The inclusion criteria for the CHF patients were a clinical diagnosis of heart failure with left ventricular ejection fraction (EF) ≤40% as measured by echocardiography. Based on EF, CHF patients were divided into moderate (EF = 26.4–32.9%) and mild (33–39.9%) CHF. Among the CHF patients, 43 patients (mild CHF = 29, moderate CHF = 14) were on anti-platelets therapy, while seven patients (mild CHF = 6, moderate CHF = 1) were on anti-coagulant therapy. Patients with heart transplantation, major surgeries, unstable angina, myocardial infarction, and stroke as well as with prolonged bed rest within eight weeks of the visit to the clinics were excluded. Hence, our study protocol is consistent with a previous study used to characterize muscle wasting in patients with CHF [10]. Data was collected from structural interviews, clinical examinations, laboratory investigations and measurements of physical parameters. This study was conducted in accordance with the declaration of Helsinki [11].

### Hand-grip strength and body composition

Hand-grip strength was measured by a digital handgrip dynamometer (CAMRY, South El Monte, CA, USA) as described before [12,13]. The participants were asked to sit down and squeeze the dynamometer with maximal strength in a smooth manner. Three attempts were performed with each hand with 60-seconds rest between each attempt and the highest value was recorded for the analysis. Appendicular skeletal muscle mass (ASM) and fat mass were calculated with the bioelectrical impedance analysis scale (RENPHO, Dubai, UAE). ASM was divided by height square to get the appendicular skeletal muscle mass index (ASMI), as described previously [14].

### Measurement of physical performance

The physical performance was assessed by the short physical performance battery (SPPB) score. This battery is composed of three timed tests: 4-m walking speed, balance, and chair-stand tests. Timed results from each test were rescored from zero (worst performers) to four (best performers). The sum of the results from the three categorized tests (ranging from 0 to 12) was used for the present analyses, as described elsewhere [15].

The walking speed was evaluated by measuring the participant’s usual gait-speed (in m/s) over a 4-m course (4-meter walk test; 4MWT). Based on the sample population quartiles, the following cut-points were used to categories the gait speed: ≤0.38 m/s, a score of 1; 0.39–0.57m/s, a score of 2; 0.58–0.76m/s, a score of 3; ≥ 0.77m/s, a score of 4.

To assess the chair-stand test, the participants were asked to stand up from a chair with their arms folded across the chest five times in a row as quickly as possible. The time needed to complete the task was recorded. The quartiles for the length of the time required for this measure was used for scoring as follows: ≥17.0s, a score of 1; 14.1–16.9s, a score of 2; 11.9–14.0s, a score of 3; and ≤ 11.8s, a score of 4.

To assess the balance test, the participants were asked to perform three increasingly challenging standing- positions: side-by-side position, semi-tandem position, and tandem position. Participants were asked to hold each position for 10s. Participants were scored as 1 if they were able to hold a side-by-side standing position for 10s, but were unable to hold a semi-tandem position for 10s; a score of 2 if they were able to hold a semi-tandem position for 10s, but were unable to hold a tandem position for more than 2s; a score of 3 if they were able to stand in tandem position for 3–9s; and a score of 4 if they were able to hold the tandem position for 10s.

### Measurements of plasma 8-isoprostanes, c-reactive proteins (CRP) and creatine kinase (CK)

We used ELISA to measure 8-isoprostanes (Cayman Chemical, Ann Arbor, MI, USA) and CRP (R&D Systems, Minneapolis, MN, USA) levels and biochemical assays to measure creatine kinase levels, as described previously [16].

### Quantification of RNA using real time-PCR

Total RNA was extracted from the blood using TRI reagent and the cDNA was prepared from 1 mg of the total RNA using iScriptTM cDNA Synthesis kit (Bio-Rad, Hercules, CA, USA) as described previously [17]. Two point five ng of cDNA samples were amplified using specific primers along with fast SYBR green master mix (Applied Biosystems, Grand Island, NY, USA). The data were analyzed using the ΔΔCt method [18].

### Quantification of circulating miRs

Bulge-LoopTM miRNA qPCR Primer Sets (RiboBio) were used to detect selected miRs expressions by qRT-PCRs with iTaq TM Universal SYBR Green Supermix (BIO-RAD) as described elsewhere [8]. Reverse transcription of the miRs into cDNA was done with the TaqMan microRNA reverse transcription kit (Thermo Fisher, Dubai, UAE) and TaqMan microRNA assays specific for selected miRs (Applied Biosystems, Thermo Fisher, Dubai, UAE) according to the manufacturer’s recommendations. Owing to several PCR sessions to analyze a high number of samples, we created a reference sample by pooling a fraction of all control and CHF samples. This reference sample was run in each PCR session to minimize the technical variability in our samples. All qRT-PCR reactions were performed in triplicate, and the signal was collected at the end of every cycle. All miRs expressions were calibrated against spike-in cel-miR-39, which lacks sequence homology to human miRs.

### Statistical analysis

Anthropometric measurements of the participants were presented using mean and standard deviation as data met the assumption for normality. Analysis of variance was used to compare groups and Pearson correlation was employed to determine the strength of the relationship between miRs levels and physical characteristics. ASM, ASMI, plasma CRP, 8-isoprostanes and CK levels were adjusted for anti-platelets therapy and daily step count, using multiple regression model. A two-sample t-test for percent was used to compare SPPB scores between the groups. Receiver operating characteristic curves were used to evaluate the sensitivity and specificity of each miR. Outliers were identified and omitted through statistical analysis. A *p*-value < 0.05 was statistically significant.

## Results

### Characteristics of the participants

The basic characteristics of the study population are summarized in Table 1. Overall, the CHF patients had lower BMI, body fats and ASMI, HGS, walking speed and daily steps count (all p < 0.05). These patients also performed poorly on SPPB scores than the healthy controls (all p < 0.05). Moreover, CHF was associated with lower plasma HDL-C levels and higher total cholesterol, LDL-C, triglycerides, 8-isoprostanes and CRP (all p < 0.05) (Table 1).

**Table 1:** Age, body composition, physical parameters, and plasma profile of healthy controls (n = 92) and patients with CHF (n = 89). Based on EF, CHF patients were divided into moderate (EF = 26.4–32.9%) and mild (33-39.9%) CHF. The numbers in parenthesis indicate the percentage of total participants for that category. Values are expressed as mean ± SD; *p < 0.05 *vs*. healthy controls, #p < 0.05 *vs*. moderate CHF. (BMI; body mass index, ASM; appendicular skeletal mass, HGS; handgrip strength, HDL; high-density lipoproteins, HbA1c; glycosylated hemoglobin, CRP; C – reactive protein).

	Healthy controls	CHF		

	n = 92	All (n = 89)	Mild (n = 63)	Moderate (n =26)

**Age at baseline (years)**	67.7 ± 5.3	64.4 ± 6.5	63.5 ± 6.1	66.5 ± 7.1
**Body composition**				
BMI (Kg/m^2^)	24.8 ± 2.8	22.2 ± 2.9*	22.5 ± 3.1*	21.5 ± 2.6*
ASM (Kg)	22.4 ± 3.1	21.1 ± 2.3	21.5 ± 2.6	20 ± 2.1*
Percent fat	29.8 ± 2.7	25.4 ± 3.1*	25.5 ± 3.2*	25.1 ± 3.3*
ASMI (Kg/m^2^)	6.8 ± 1.8	6.2 ± 1.4*	6.3 ± 1.44	6.1 ± 1.31
**Physical capacity**				
HGS (Kg)	23.5 ± 3.2	18.6 ± 3.3*	19.5 ± 3*	16.4 ± 3.6*#
HGS/ASM	1.05 ± 0.21	0.88 ± 0.18*	0.90 ± 0.15*	0.81 ± 0.21*#
4-meter walking Speed (m/s)	1.18 ± 0.17	0.97 ± 0.13*	1.01 ± 0.11*	0.87 ± 0.19*
Daily steps count	5,211 ± 1113	3,427 ± 838*	3,708 ± 813*	2,746 ± 928*
**SPPB score**				
12	44 (47.8)	4 (4.4)*	4 (6.3)*	0
11	18 (19.5)	8 (8.9)*	6 (9.5)*	2 (7.7)
10	15 (16.3)	22 (24.7)*	17 (27)	5 (19.2)
9	7 (7.6)	33 (37.1)*	24 (38)*	9 (34.6)*
8	5 (5.4)	17 (19.1)*	11 (17.5)*	6 (23.1)*
7	3 (3.2)	4 (4.5)	1 (1.6)	3 (11.5)
6	0	1 (1.1)	0	1 (3.8)
**Plasma profile**				
Total Cholesterol	187.4 ± 25.4	236.3 ± 39.8*	232.4 ± 40.5*	246.5 ± 37.9*
HDL-C (mg/dl)	40.2 ± 2.8	37.1 ± 2.7*	37.4 ± 2.8	36.4 ± 2.2*
LDL-C (mg/dl)	97.8 ± 14.9	149.8 ± 29.2*	146.3 ± 24.9*	154.4 ± 33.5*
Triglycerides (mg/dl)	144.3 ± 26.3	197.6 ± 39.3*	189.4 ± 35.5*	216.5 ± 42.3*
HbA1c (%)	5.81 ± 0.11	5.93 ± 0.17	5.89 ± 0.15	6.04 ± 0.19
8-isoprostanes (pg/ml)	51.6 ± 9.7	106.9 ± 28.5*	102.4 ± 26.2*	116.7 ± 32.6*#
CRP (mg/dl)	0.15 ± 0.03	0.32 ± 0.08*	0.31 ± 0.07*	0.34 ± 0.07*
Creatine kinase (IU/L)	182.3 ± 19.7	291.4 ± 38.7	281.7 ± 34.6	315.3 ± 37.4*

### Levels of circulating miRs and the markers of inflammation and oxidative stress

**Figure 1:**
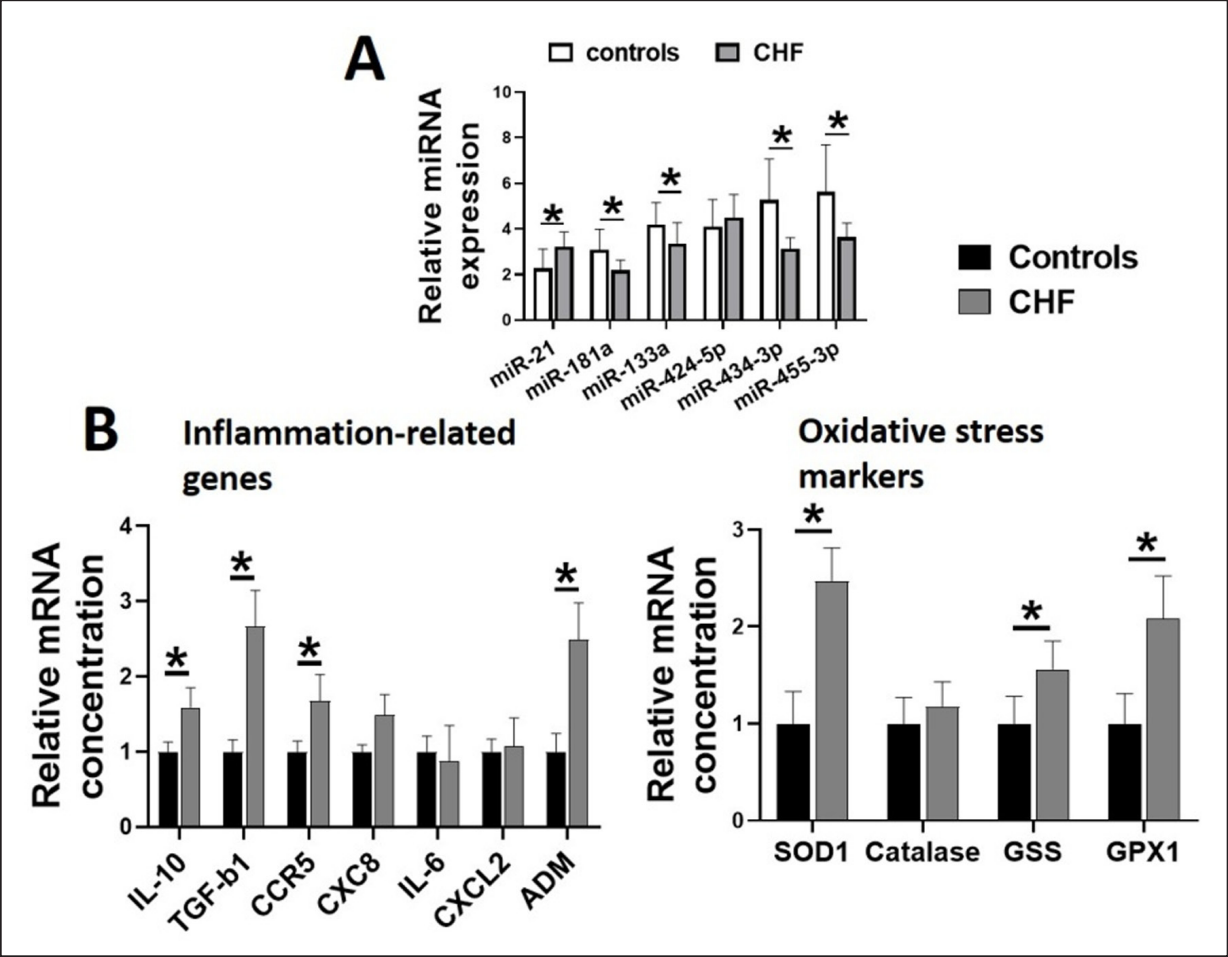
Relative expressions of the plasma miRs **(A)** and the markers of inflammation and oxidative stress **(B)** in the healthy controls (n = 92) and the patients with CHF (n = 89). Values are expressed as mean ± SEM, *p < 0.05 *vs*. the healthy controls. (Interleukin-10, IL-10; Transforming growth factor-beta 1, TGF-b1; c-c motif chemokine receptor 5, CCR5; c-x-c motif chemokine ligand 8, CXCL-8; Interleukin-6, IL-6; c-x-c motif chemokine ligand 2, CXCL2; adrenomedullin, ADM; superoxide dismutase-1, SOD1; glutathione synthetase, glutathione peroxidase-1 GPX1).

We next measured the levels of selected circulating miRs and mRNA markers of inflammation and oxidative stress in the healthy controls and CHF patients. Among the miRs, miR-21 levels were upregulated while miR-181a, miR-133a, miR-434-3p and miR-455-3p levels were downregulated in the patients with CHF (all p < 0.05), when compared to control group (Figure 1A).

Since these miRs also regulate inflammation and cellular redox environment, we next measured the plasma mRNA markers of inflammation and oxidative stress. We found higher plasma levels of pro-inflammatory cytokines IL-10, TGF-b1 and ADM, and antioxidant enzymes SOD1, catalase, GSS and GPX1 in the patients with CHF (all p < 0.05), when compared to controls (Figure 1B). We further confirmed the increased inflammation and oxidative stress in CHF patients by reporting elevated plasma levels of CRP (107%, p < 0.05) and 8-isoprostanes (113%, p < 0.05) in CHF patients than healthy controls (Table 1).

### Correlation of miRs with physical capacity and sarcopenia indexes

**Figure 2:**
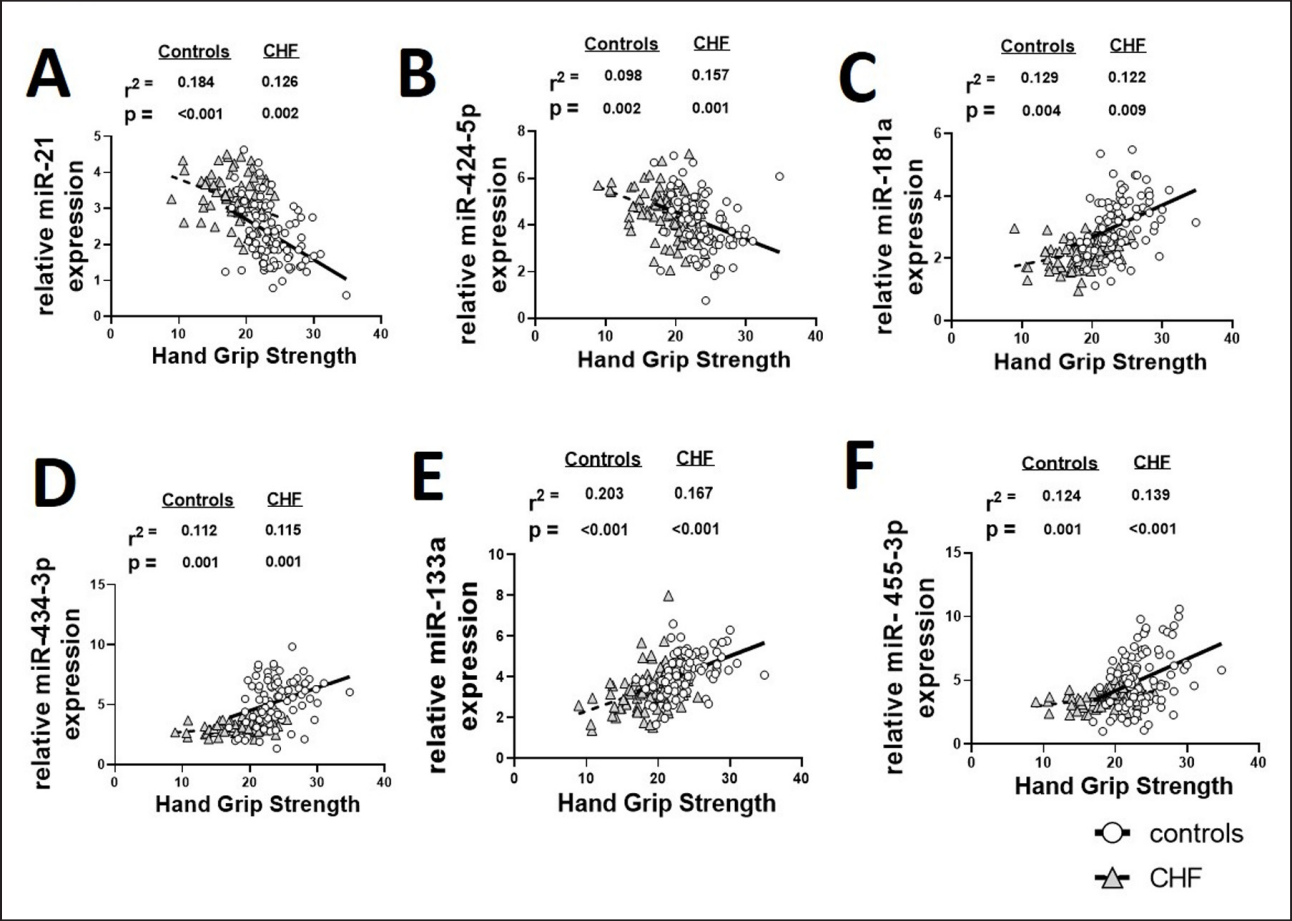
Linear regression analysis of the relationships of plasma miRs with hand-grip strength (HGS) in healthy controls (n = 92) and patients with the CHF (n = 89).

European working group on sarcopenia in older people characterizes sarcopenia with low muscle strength and mass, and low gait speed [19]. We used HGS, ASM, ASMI and 4MWT as the indexes for the detection of sarcopenia. All six miRs showed statistically significant correlations with HGS in healthy controls and patients with CHF (Figure 2) (all p < 0.05).

Among the individual miRs, miR-133a showed the strongest correlation with HGS in controls (r^2^ = 0.203, p < 0.001) and CHF group (r^2^ = 0.167, p < 0.001). We also found significant correlations of other miRs with ASM in both study cohorts except miR-21 and miR 434-3p in healthy controls and miR-181a in CHF group (all p < 0.05). However, when ASM was adjusted for height square (ASMI), a significant correlation was only found with miR-434-3p (in healthy controls), miR-133a (in CHF group) and miR-455-3p (in healthy controls and CHF groups) (all p < 0.05). On the other hand, 4MWS showed significant correlation with miR-133a and miR-455-3p (healthy controls) and all the miRs except miR-455-3p (in patients with CHF) (all p < 0.05) (Table 2).

**Table 2:** Correlations coefficients (r^2^) of circulating mi-RNAs with absolute and adjusted (for anti-coagulant therapy and daily step count) HGS, ASM, ASMI and 4MWS in healthy controls (n = 92) and participants with CHF (n = 89), * p < 0.05.

	MiR-21	MiR-181a	miR-133a	MiR-424-5p	MiR-434-3p	MiR-455-3p

**HGS**						
Healthy	0.184*	0.129*	0.203*	0.098*	0.112*	0.124*
CHF	0.126*	0.122*	0.167*	0.157*	0.115*	0.139*
**ASM**						
Healthy	0.126	0.204*	0.136*	0.116*	0.098	0.321*
CHF	0.246*	0.171	0.15*	0.112	0.204*	0.24*
**ASMI**						
Healthy	0.086	0.059	0.095	0.058	0.118*	0.128*
CHF	0.034	0.018	0.273*	0.104*	0.081	0.094*
**4MWS**						
Healthy	0.065	0.049	0.083*	0.067	0.096	0.113*
CHF	0.089	0.177*	0.095*	0.137*	0.128*	0.098
**Adjusted HGS**						
Healthy	0.131*	0.103*	0.184*	0.075*	0.063	0.103*
CHF	0.109*	0.128*	0.181*	0.139*	0.078	0.164*
**Adjusted ASM**						
Healthy	0.107	0.225*	0.124*	0.096	0.109*	0.285*
CHF	0.155*	0.104	0.157*	0.135*	0.188*	0.256*
**Adjusted ASMI**						
Healthy	0.075	0.068	0.064	0.086	0.129*	0.155*
CHF	0.046	0.024	0.183*	0.169*	0.108*	0.109*
**Adjusted 4MWS**						
Healthy	0.098*	0.055	0.074	0.075	0.122*	0.205*
CHF	0.084	0.205*	0.108*	0.103*	0.187*	0.129*

### Circulating miRs accurately identify accelerated sarcopenia in CHF

**Figure 3:**
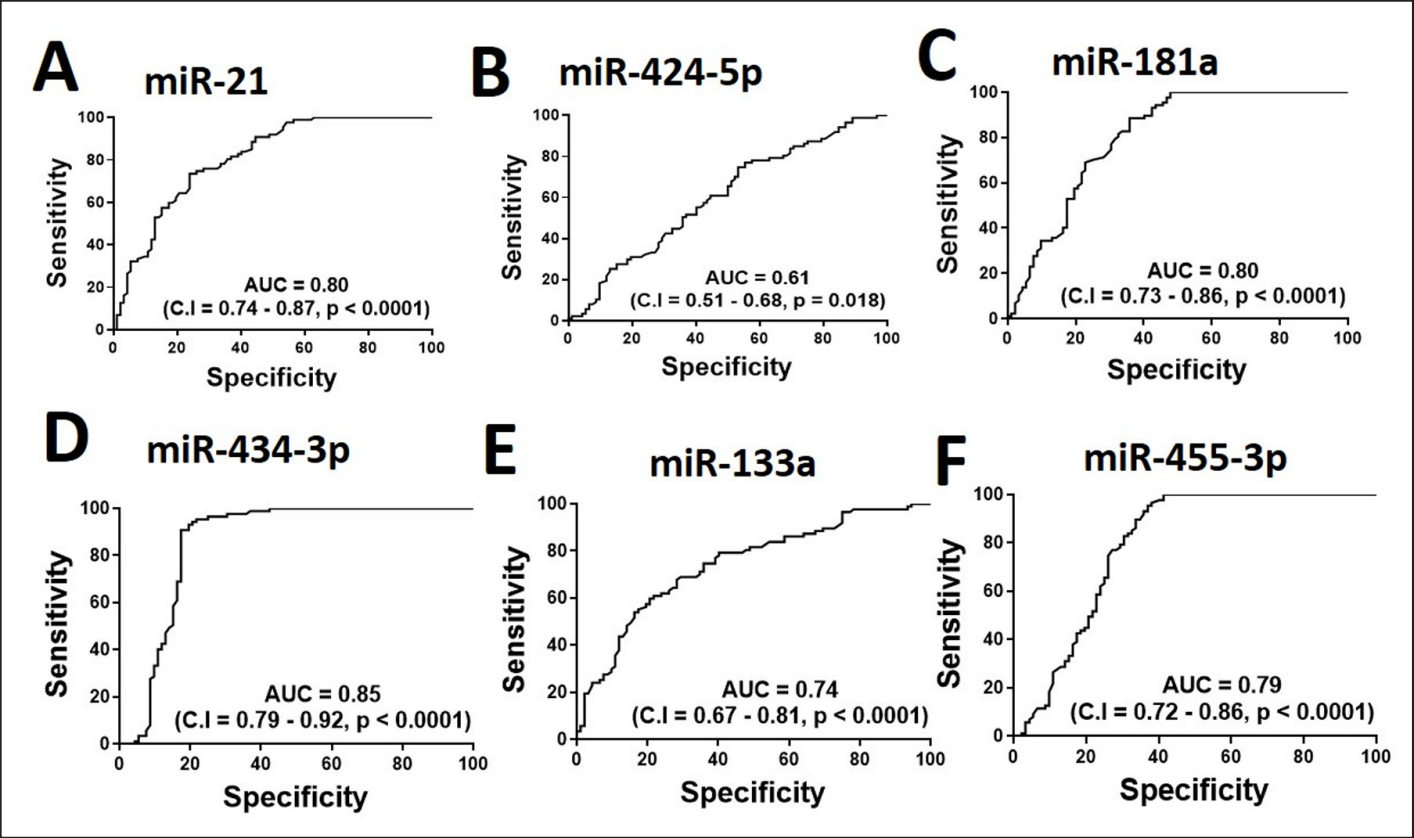
ROC curve illustrating sensitivity and specificity of the plasma miRs in discriminating healthy controls (n = 92) from the patients with CHF (n = 89).

We next tested the diagnostic potentials of circulating miRs in CHF patients with sarcopenia, using receiver-operative characteristic (ROC) curves (Figure 3). While all miRs displayed significant values, we found that the miR-424-5p exhibited the highest area under the curve (AUC = 0.85), followed by miR-21 and miR-181a (both AUC = 0.80). On the other hand, miR-424-5p displayed the lowest but still significant value (AUC = 0.61) (Figure 3).

### Correlation of circulating miRs with markers of inflammation, oxidative stress and muscle damage

Given the importance of the selected miRs in the regulation of inflammation, oxidative stress, and muscle health, we next determined the correlations of plasma 8-isoprostanes, CRP and CK levels with circulating miRs expressions. Plasma 8-isoprostanes levels, a marker of oxidative stress showed significant correlation with miR-455-3p (healthy controls), miR-181a (CHF group) and miR-424-5p (in both groups) (all p < 0.05) (Table 3) [20]. On the other hand, plasma GRP level, a marker of generalized inflammation showed significant correlation with miR-455-3p (healthy controls), miR-21, miR-424-5p, miR-434-3p (CHF group), miR-133a and miR-455-3p (in both groups) (all p < 0.05). Plasma CK levels are increased in muscle injury and were correlated with miR-181a (healthy controls), miR-21 (CHF group), miR-133a, miR-424-5p, miR434-3p and miR-455-3p (in both groups) (all p < 0.05) (Table 3).

**Table 3:** Correlations coefficients (r^2^) of circulating mi-RNAs with absolute and adjusted (for anti-coagulant therapy and daily stem count) plasma 8-isoprostanes and CRP levels in healthy controls (n = 92) and participants with CHF (n = 89), * p < 0.05.

	MiR-21	MiR-181a	miR-133a	MiR-424-5p	MiR-434-3p	MiR-455-3p

**8-isoprostanes**						
Healthy	0.094	0.105	0.095	0.239*	0.053	0.079*
CHF	0.079	0.185*	0.119	0.179*	0.076	0.068
**CRP**						
Healthy	0.134	0.046	0.149*	0.028	0.071	0.139*
CHF	0.169*	0.108*	0.253*	0.131*	0.105*	0.261*
**Plasma CK**						
Healthy	0.085	0.103*	0.187*	0.379*	0.158*	0.141*
CHF	0.129*	0.094	0.353*	0.413*	0.224*	0.178*
**Adjusted 8-isoprostanes**						
Healthy	0.081	0.129*	0.071	0.204*	0.059	0.097*
CHF	0.098	0.151*	0.089	0.191*	0.071	0.074
**Adjusted CRP**						
Healthy	0.155*	0.053	0.163*	0.036	0.084	0.128*
CHF	0.188*	0.094*	0.204*	0.152*	0.118*	0.218*
**Adjusted Plasma CK**						
Healthy	0.104	0.129*	0.166*	0.398*	0.144*	0.166*
CHF	0.144*	0.104	0.308*	0.367*	0.219*	0.152*

## Discussion

The following major findings emerge from our study: 1) Patients with CHF had lower plasma miR-21 and higher plasma miR-181a, miR-133a, miR-434-5-p and miR-455-3p levels than the healthy controls. 2) Among the plasma miRs, miR-133a showed the strongest correlations with HGS, while miR-133a, miR-434-3p and miR-455-3p showed the strongest correlation with ASMI. 3) miR-434-3p shows the highest area under the curve in the testing for sensitivity and specificity for CHF.

Sarcopenia is a senile syndrome with very complex pathogenesis. Several diseases can induce muscle atrophy and weakness independent of aging, indicating the susceptibility of skeletal muscle to systemic morbidities. Patients with CHF have a higher prevalence of sarcopenia than the age-matched elderly without CHF [10]. Further, the rate of muscle decline is faster in these patients, leading to a further reduction in physical capacity and cardiac function [21,22]. Circulating miRs hold a great potential as biomarkers of muscle decline in CHF. However, several limitations reduce the diagnostic potentials of miRs in sarcopenia. Indeed, inaccurate normalization methods and smaller sample pools can partly account for the discrepancy in the literature. Further, the variability in clinical conditions of CHF patients can also hamper the reproducibility of the data. We analyzed more than 180 subjects in this study, which is a considerable sample size to draw stronger conclusions. Stringent normalization methods and acceptance criteria were used to further enhance the confidence in our data.

Several of the miRs showed significant correlations, albeit biologically weak, with the clinical parameters of sarcopenia, supporting the hypothesis that the altered plasma expressions of these miRs are specifically related to muscle damage. Among the various miRs investigated here, miR-133a is primarily produced by skeletal muscle and has a role in muscle differentiation and proliferation [23]. Aging is associated with an upregulation of miR-133a expression in skeletal muscle [5]. However, it is not known whether the plasma miR-133a levels are due to active secretion by skeletal muscles or passive release from the damaged myofibers. We found a strong association between plasma miR-133a and CK levels, which indicates that the muscle damage is at least partly contributing to plasma miR-133a levels. Indeed, the CHF results in skeletal muscle damage and necrosis [24], which can facilitate passive release of miR-133a into circulation. Other miRs investigated here are released by multiple tissues including nervous tissues, testes, epididymis and spleen (https://ccb-web.cs.uni-saarland.de/tissueatlas/). Thus, the perturbations in plasma levels of these miRs may represent the generalized clinical condition of the CHF patients rather than muscle-specific disorders. This is not surprising, considering the multi-system effects of CHF [25].

We found considerable discordance between the ROC analysis and the predictive power of miRs in diagnosing sarcopenia. For example, miR-434-3p had the highest AUC on the ROC curve but performed relatively poorly in diagnosing loss of muscle mass, strength, and physical capacity. miR-434-3p is importantly expressed in both the cardiac and skeletal muscle and has a pathological role in both tissues [6,26]. For example, the expression of miR-434-3p is increased in the failing heart in heart diseases [26]. Plasma miR-434-3p levels also reflect muscle atrophy during aging [6]. Thus, it is possible that the different contributions of skeletal and cardiac muscles to the circulating miR-434-3p levels account for different discriminating power in assessing cardiac and skeletal muscle health.

We did not perform a functional analysis to investigate the target genes of selected miRs. However, to gain insight into the possible mechanisms by which these miRs regulate muscle health, we used Target Scan (http://www.targetsca.org/), an online database for predicting miR targets. We found between 400 to 1900 targets for miR-121, miR-424-5p, miR-133a, miR-455-3p and miR-181a, while miR 434-3p was not in the Target Scan database. We next used the Kyoto encyclopedia of genes and genomes pathway analysis (http://www.kegg.jp/kegg/pathway.html) with a cut-off criteria of a target gene number > 2 and p < 0.05. We found the involvement of pathways associated with skeletal muscle calcium signaling, protein synthesis, MAPK signaling, unfolded protein response, ERK and FOXO signaling. We also found a role of miR-434-3p in regulating age-related apoptosis in the skeletal muscle [27].

The expressions of selected miRs in the erythrocytes is minimal or non-existence, which largely rules out potential distorting effects of hemolysis [28]. Additionally, large sample size and stringent normalization and acceptance criteria are the strengths of this study. Nevertheless, some limitations must be acknowledged. The study includes men only so we cannot dissect these effects in a gender-specific manner. This is important since a gender-bias is recognized in the expressions of some miRs including miR-133 [29,30]. We did not measure the quadriceps strength, which is also used apart from HGS as a sarcopenia index [31]. The selective survival of the CHF patients before inclusion in this study is worth consideration. The pathophysiological mechanism(s) by which the selected miRs contribute to sarcopenia phenotype in CHF remains elusive. We also did not characterize the relative contributions of skeletal and cardiac muscles and other tissues to the circulating miRs levels. We also do not know if the detected circulating miRs are primarily released via active mechanisms or due to tissue damage.

Taken together, we show that the plasma miR-133a, miR-434-3p and miR-455-3p can potentially act as novel biomarkers of skeletal muscle health and physical capacity in CHF. Their plasma levels can also provide information on functional compromise and risk stratification in sarcopenia with CHF. Additional studies with a more mechanistic approach are required to characterize the functions of these miRs in sarcopenia.

## Data Accessibility Statement

Raw data from the patient cohorts cannot be shared due to the intellectual property rights but are available from the corresponding author on reasonable request.
